# Interrelationships among whole-body skeletal muscle mass, masseter muscle mass, oral function, and dentition status in older Japanese adults

**DOI:** 10.1186/s12877-021-02552-9

**Published:** 2021-10-20

**Authors:** Masanori Iwasaki, Hirohiko Hirano, Keiko Motokawa, Maki Shirobe, Ayako Edahiro, Yuki Ohara, Hisashi Kawai, Motonaga Kojima, Shuichi Obuchi, Hiroshi Murayama, Yoshinori Fujiwara, Kazushige Ihara, Shoji Shinkai, Akihiko Kitamura

**Affiliations:** 1 Tokyo Metropolitan Institute of Gerontology, 35-2 Sakae-cho, Itabashi-Ku, Tokyo, 173-0015 Japan; 2 Department of Physical Therapy, University of Tokyo Health Sciences, 4-11, Ochiai, Tama City, Tokyo 206-0033 Japan; 3 Department of Social Medicine, Hirosaki University Graduate School of Medicine, 5 Zaifu-cho, Hirosaki City, Aomori 036-8562 Japan; 4 School of Nutritional Sciences, Kagawa Nutrition University, 3-9-21 Chiyoda Sakado, Saitama, 350-0288 Japan

**Keywords:** Cross-sectional study, Epidemiology, Masticatory muscles, Geriatrics, Oral health

## Abstract

**Background:**

Generalized loss of skeletal muscle mass (SMM) may modulate or otherwise affect the loss of masseter muscle mass and be responsible for low masseter muscle performance and strength (i.e., low oral function). Moreover, dentition status can affect oral function independent of the muscle state. This cross-sectional study aimed to simultaneously investigate the relationships among whole-body SMM, masseter muscle mass, oral function (masseter muscle performance and strength), and dentition status in 1349 Japanese adults (mean age = 73.6 years).

**Methods:**

We determined the estimated masseter muscle mass (e-MMM) based on morphological measurements of the masseter muscle. Masseter muscle performance was assessed via masticatory performance evaluation scores using gum, and strength was assessed as the maximal occlusal force. Dentition status was assessed as the number of functional teeth. SMM was measured by bioelectrical impedance analysis. Structural equation modeling stratified by sex was employed to investigate associations among SMM, e-MMM, gum score, occlusal force, and number of functional teeth.

**Results:**

The direct path from SMM to e-MMM was statistically significant, as was the direct path from e-MMM to oral function (gum score and maximum occlusal force) for both sexes. We additionally confirmed that SMM indirectly affected gum score and maximum occlusal force via e-MMM (men; standardized coefficient [95% CI] = 3.64 [1.31 to 5.96] for maximum occlusal force and 0.01 [0.01 to 0.02] for gum score, women; 2.01 [0.38 to 3.81] for maximum occlusal force and 0.01 [0.002 to 0.01] for gum score). The number of functional teeth had direct effects on e-MMM, gum score, and maximum occlusal force.

**Conclusions:**

Low SMM was significantly indirectly associated with poor oral function through a low masseter muscle mass, and dentition status was independently associated with oral function.

## Background

Oral function is important for longevity. Previous studies have reported that poor oral function, including low masticatory performance and low occlusal force, is associated with cardiovascular disease, frailty, and mortality in older adults [1–3] and that this association is partly mediated by poor dietary intake and malnutrition [4, 5]. The masticatory muscles play an important role in oral function, especially mastication [6]. In particular, the masseter muscle is significant for mastication [7, 8].

Because the oral cavity is an organ within the body system, generalized loss of skeletal muscle mass (SMM) may either affect or modulate the loss of masseter muscle mass [9–13]. A decrease in muscle mass causes decreases in muscle performance and strength [14]. Therefore, a decrease in masseter muscle mass can be responsible for low masseter muscle performance (i.e., low masticatory performance) and low masseter muscle strength (i.e., low occlusal forces). Overall, it is hypothesized that the whole-body SMM has indirect effects on the function and strength of the masseter muscles through the masseter muscle mass.

In addition to masseter muscle mass, dentition status, including the status of natural teeth and dental prostheses (e.g., removable dentures, dental bridges, and dental implants), is a significant factor influencing masticatory function in older adults [15, 16]. It has also been shown that tooth loss and the absence of dental prostheses are associated with decreased masseter muscle thickness, which is considered a consequence of muscle atrophy due to low activity and disuse of the masseter muscles [9, 17].

To date, the relationships among whole-body SMM; masseter muscle mass, performance, and strength; and dentition status in older adults have not been fully investigated. The aim of this study was to simultaneously analyze the relationships among whole-body SMM, masseter muscle mass, oral function (masseter muscle performance and strength), and dentition status in community-dwelling older adults. Our priori hypotheses were as follows: 1) low whole-body SMM is associated with low masseter muscle mass, which mediates the relationship between low whole-body SMM and poor oral function; and 2) dentition status is associated with masseter muscle mass, performance, and strength independent of whole-body SMM.

## Methods

### Study population

The current cross-sectional investigation is a secondary analysis of integrated data from two cohort studies: the Otassha Study and the Kusatsu Longitudinal Study. Both studies included community-dwelling adults aged ≥65 years. The designs and protocols of the studies have been described in detail elsewhere [18, 19]. The variables used for masseter muscle mass estimation were assessed in 2012 for the Otassha Study and in 2014 for the Kusatsu Longitudinal Study. The data collected at the two time points were integrated and used for the present study.

Individuals with missing data were excluded. The Otassha Study and Kusatsu Longitudinal Study were conducted in full accordance with the ethical principles of the Declaration of Helsinki and were approved by the Ethics Committee of the Tokyo Metropolitan Institute of Gerontology. All participants provided written informed consent prior to participating.

### Assessment of oral health status

Oral health assessments, including determination of the estimated masseter muscle mass (e-MMM), determination of the dentition status, measurement of the maximal occlusal force, and assessment of masticatory function, were carried out by six qualified dentists (H.H., A.E., Yoshiko Motohashi, Daisuke Takagi, Masaharu Murakami, and Kento Umeki). They performed oral health assessments in both cohorts (i.e., the Otassha Study and Kusatsu Longitudinal Study).

Prior to examinations, calibrations were performed at the Tokyo Metropolitan Institute of Gerontology by examining volunteer subjects. Measurements of the vertical length, width (horizontal length), and thickness of the masseter muscle yielded intraclass correlation coefficients > 0.8. For measurement of maximal occlusal force, the gold standard examiner (H.H.), a board-certified trainer of the Japanese Society of Gerodontology, confirmed that the five other examiners had placed the pressure-sensitive sheets at the correct position between the upper and lower dental arches. For the assessment of masticatory function, five examiners obtained a minimum kappa value of 0.9 compared to the gold standard obtained by another examiner (H.H.). All examiners obtained intra-examiner kappa values > 0.9 based on the results repeated assessments. In the kappa calculations, scores that were exactly equal indicated agreement.

### Estimation of the masseter muscle mass

The e-MMM was obtained by multiplying the vertical length, width, and thickness of the masseter muscle, which were measured according to a published protocol [8]. The right masseter muscle was the objective of the assessment, and the detailed protocol was as follows. After the masseter muscle was palpated to determine its position, the length and width of the masseter muscle were measured using calipers. First, the vertical length of the masseter muscle was measured as the distance from the center of the muscle’s origin at the inferior border of the zygomatic arch to the center of the muscle at the inferior border of the mandible. Then, the width of the central region of the masseter muscle was measured as the horizontal length of the muscle.

The thickness of the masseter muscle was measured using an ultrasound measuring device (Miru-Cube; Global Health Co., Ltd., Kanagawa, Japan). After the masseter muscle was palpated, its thickness was measured with the device’s probe, which was placed parallel to the mandibular plane on the masseter muscle along the extension line of the oral angle while the participants were in a relaxed state. The measurement was performed twice, and the mean value was used for the study.

### Determination of the dentition status

Trained dentists assessed each tooth as present, missing with replacement by a dental prosthesis, or missing without replacement. During the assessment, there was no distinction between replacement of missing teeth by fixed dental prostheses (i.e., dental bridges or dental implants) and replacement of missing teeth by removable dental prostheses (i.e., dentures). The number of natural teeth was defined as the number of remaining teeth, excluding residual roots. The number of functional teeth was defined as the number of natural teeth, artificial teeth in dentures, pontics on bridges, and implants [8]. The number of functional teeth was considered a measure of the dentition status [20].

### Measurement of the maximal occlusal force

The maximal occlusal force was measured using pressure-sensitive sheets (Dental Prescale; Fuji Film Co., Tokyo, Japan). The participants were asked to clench their jaws with maximal force in the intercuspal position for 3 s with pressure-sensitive sheets placed between the upper and lower dental arches. Participants who routinely used removable dentures underwent examination while wearing their dentures to ensure optimum oral function [21, 22]. The maximal occlusal force was calculated after the sheet was scanned with an image scanner (Occluzer, FPD-707; Fuji Film Co., Tokyo, Japan) [23].

### Assessment of masticatory function

Masticatory function was assessed using gum for evaluating masticatory performance (XYLITOL, 70 mm × 20 mm × 1 mm, 3.0 g; Lotte, Saitama, Japan). The participants were asked to chew a piece of gum for 2 min in the same manner that they usually chew food. Participants who routinely used removable dentures underwent examination while wearing their dentures to ensure optimum oral function [21, 24]. Immediately after the participants had chewed the gum, the color of the chewed gum was evaluated using a color chart with five color gradations. Each participant was assigned a score ranging from 1 to 5, with higher scores indicating better masticatory function. The validity and reliability of masticatory functional assessment using masticatory performance evaluations based on gum with a color scale were reported in a previous study [25]. There was a strong correlation (Pearson’s correlation coefficient = 0.98) between the color scale scores obtained and the values measured by a colorimeter [25].

### Measurement of the SMM

The SMM was measured by bioelectrical impedance analysis using a body composition analyzer (InBody 720; Biospace Co., Ltd., Seoul, Korea) with the study participants in the standing position. Measurement was performed following the instructions of the device manufacturer. In addition, as recommended in the device manual, participants wiped the bottom of their feet with an electrolyte tissue before measurement.

### Data collection for other variables

Data on the participants’ age, sex, smoking status, alcohol consumption, and physical activity level were obtained through a self-administered questionnaire. Regarding the smoking status, the participants were considered current smokers or nonsmokers. Regarding alcohol consumption, the participants were considered daily drinkers or nondrinkers. The physical activity level was defined as low if the participant answered “no” to the question “Do you engage in physical exercise or sports?”

Anthropometric measures included weight and height. The body mass index (BMI) was calculated by dividing weight in kilograms by height squared in meters. BMI values < 18.5 kg/m^2^ were defined as low.

Each participant was allocated 1 point for being a current smoker, 1 point for being a daily drinker, 1 point for having a low physical activity level, and 1 point for having a low BMI. A score of 0 was assigned for these factors if the participants did not meet the above specifications. A composite lifestyle score (0–4 points) was generated by adding the points for each of the four factors, with higher scores indicating an unhealthier lifestyle.

Comorbidity factors, including stroke, heart disease, diabetes, osteoporosis, and respiratory diseases, were identified through medical interviews. The number of comorbidities was determined.

### Statistical analyses

Analyses were performed with the statistical software package STATA, version 16.1 (StataCorp, College Station, USA). The current investigation used integrated data from two cohort studies. The random effects of the cohort were tested by applying a multilevel model.

We employed T-tests, Mann-Whitney U-tests, or chi-squared tests when appropriate to compare the study population characteristics of the men and women.

The Spearman correlation coefficient was used to assess the relationships among the variables (SMM, e-MMM, masticatory variables, functional teeth, age, lifestyle, and comorbidities).

We employed a structural equation modeling (SEM) to examine relationships and predictions among the SMM, e-MMM, masticatory variables, and dentition status. No latent variables were included. We started the analysis with the model based on our hypothesis, as discussed in an earlier section. The model was iteratively modified by adding a path link or including demographic and health-related variables. The optimal model was selected as the model with the lowest Bayesian information criterion (BIC) [26]. In addition to the BIC, we used the comparative fit index (CFI) and the root mean square error of approximation (RMSEA) as model fit indices. We considered a model to have an acceptable fit when it had a CFI of > 0.9 and an RMSEA of < 0.1 [27]. The final model was selected based on the theoretical meaningfulness of the relationship and the statistical significance of the path coefficient. Bias-corrected standard errors of the effects were calculated using a bootstrapping approach with 1000 resamples. The level of significance was set to 0.05.

We additionally employed SEMs for subgroup analyses, with participants stratified according to age (< 75 and ≥ 75 years old).

## Results

### Study population characteristics

**Fig. 1:**
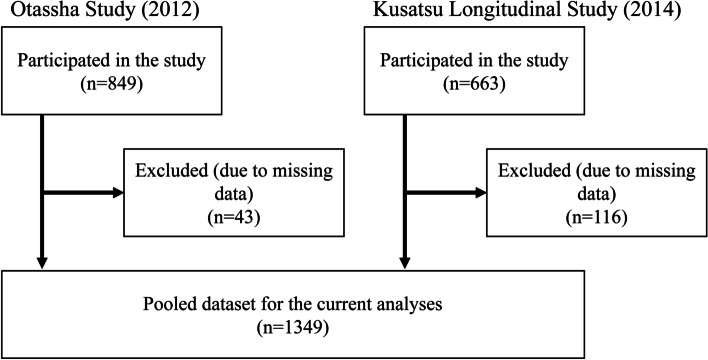
The selection of data sources

In total, 1512 individuals (849 from the Otassha Study 2012 and 663 from the Kusatsu Longitudinal Study 2014) participated in the survey. We excluded 163 of these individuals (47 from the Otassha Study 2012 and 116 from the Kusatsu Longitudinal Study 2014) because of missing data. The remaining 1349 individuals (572 men and 809 women, with a mean [standard deviation] age of 73.6 [5.8] years) were included in the present analysis (Fig. 1).

Table 1 presents the characteristics of the participants according to sex. The male participants had larger e-MMM, masseter muscle length and masseter muscle thickness values than did the female participants. In addition, males had larger occlusal forces and higher gum scores and SMMs than did females. Regarding lifestyle factors, the proportions of current smokers and daily drinkers were higher, the level of physical activity was lower, the proportion of participants with a low BMI was lower, and the combined score indicating an unhealthier lifestyle was higher among males than females. Regarding comorbidities, stroke, heart disease, respiratory disease, and cancer were observed more frequently among males, while osteoporosis was observed more frequently among females.

**Table 1:** Study population characteristics according to sex

	Total (***n*** = 1349)	Women (***n*** = 791)	Men (***n*** = 558)	***p***-value
Oral variables				
e-MMM (cm^3^), mean (s.d.)^a^	22.2 (9.1)	20.1 (8.0)	25.3 (9.7)	< 0.01
Vertical length of the MM (mm), mean (s.d.)^a^	49.8 (7.2)	48.0 (6.5)	52.2 (7.3)	< 0.01
Horizontal length of the MM (mm), mean (s.d.)^a^	38.8 (7.0)	37.4 (6.7)	40.6 (7.1)	< 0.01
Thickness of the MM (mm), mean (s.d.)^a^	11.3 (3.0)	11.0 (2.9)	11.8 (3.0)	< 0.01
Maximum occlusal force (N), median (IQR)^b^	345 (173–595)	315 (157–537)	409 (207–674)	< 0.01
Gum score, median (IQR)^bd^	4 (3–4)	4 (3–4)	4 (3–4)	< 0.01
*n* of natural teeth, median (IQR)^b^	21 (9–26)	21 (9–26)	20 (9–26)	0.57
*n* of functional teeth, median (IQR)^b^	28 (27–28)	28 (27–28)	28 (26–28)	0.68
Age, mean (s.d.)^a^	73.6 (5.8)	73.3 (5.6)	74.0 (6.0)	0.022
SMM (kg), mean (s.d.)^a^	21.0 (4.5)	18.2 (2.2)	25.1 (3.6)	< 0.01
Lifestyle factors				
Current smoking, *n* (%)^c^	145 (10.7%)	50 (6.3%)	95 (17.0%)	< 0.01
Daily alcohol intake, *n* (%)^c^	283 (21.0%)	77 (9.7%)	206 (36.9%)	< 0.01
Low physical activity, *n* (%)^c^	301 (22.3%)	165 (20.9%)	136 (24.4%)	0.13
Body weight (kg), mean (s.d.)^a^	55.6 (10.3)	50.9 (8.1)	62.3 (9.4)	< 0.01
Height (cm), mean (s.d.)^a^	155.3 (8.6)	150.0 (5.7)	162.7 (6.2)	< 0.01
BMI (kg/m^2^), mean (s.d.)^a^	23.0 (3.3)	22.6 (3.5)	23.5 (3.0)	< 0.01
BMI < 18.5 kg/m^2^, *n* (%)^c^	96 (7.1%)	76 (9.6%)	20 (3.6%)	< 0.01
Combined score for an unhealthy lifestyle, median (IQR)^b^	0 (0–1)	0 (0–1)	1 (0–1)	< 0.01
Comorbidities				
Stroke, *n* (%)^c^	97 (7.2%)	42 (5.3%)	55 (9.9%)	< 0.01
Heart disease, *n* (%)^c^	179 (13.3%)	85 (10.7%)	94 (16.8%)	< 0.01
Diabetes, *n* (%)^c^	180 (13.3%)	75 (9.5%)	105 (18.8%)	< 0.01
Osteoporosis, *n* (%)^c^	161 (11.9%)	155 (19.6%)	6 (1.1%)	< 0.01
Respiratory disease, *n* (%)^c^	39 (2.9%)	17 (2.1%)	22 (3.9%)	0.05
Cancer, *n* (%)^c^	164 (12.2%)	82 (10.4%)	82 (14.7%)	0.02
Number of comorbidities, median (IQR)^b^	0 (0–1)	0 (0–1)	0 (0–1)	0.44

*e-MMM* estimated masseter muscle mass, *MM* masseter muscle, *IQR* interquartile range, *s.d.* standard deviation, *SMM* skeletal muscle mass

^a^T-tests were employed to compare the study population characteristics between men and women

^b^Mann-Whitney U-tests were employed

^c^Chi-squared tests were employed

^d^Somers’ D (median difference between men and women) = 0.11, 95% confidence interval = 0.05–0.17 (reference group = women)

Because significant differences in the SMM and e-MMM were observed between men and women, the following analyses were performed according to sex.

### Associations among SMM, e-MMM, masticatory variables, functional teeth, age, lifestyle, and comorbidities

Table 2 shows the correlations among SMM, e-MMM, masticatory variables, functional teeth, age, lifestyle, and comorbidities according to sex. A likelihood-ratio test comparing the two-level model (i.e., including random effects due to the cohort field) and single-level model (i.e., without random effects) was not significant for our data (*p* = 0.45). No significant random effects due to the cohort field were observed; therefore, a single-level model was used.

**Table 2:** Correlation between the SMM, e-MMM, masticatory variables, age, and health variables according to sex

		**Men**						
**Variables**		**1**	**2**	**3**	**4**	**5**	**6**	**7**
1	SMM							
2	e-MMM	0.24*						
3	Maximum occlusal force	0.21*	0.24*					
4	Gum score	0.24*	0.25*	0.49*				
5	Functional teeth	0.04	0.08	0.16*	0.17*			
6	Age	−0.41*	−0.12*	−0.18*	−0.17*	−0.07		
7	Combined score for an unhealthy lifestyle	−0.01	− 0.09*	− 0.05	− 0.12*	0.02	− 0.12*	
8	Number of comorbidities	− 0.06	− 0.04	− 0.14*	− 0.08	− 0.02	0.13*	− 0.05
		**Women**						
**Variables**		**1**	**2**	**3**	**4**	**5**	**6**	**7**
1	SMM							
2	e-MMM	0.19*						
3	Maximum occlusal force	0.16*	0.14*					
4	Gum score	0.21*	0.13*	0.46*				
5	Functional teeth	−0.05	0.03	0.07	0.12*			
6	Age	−0.31*	−0.08*	−0.23*	−0.22*	0.05		
7	Combined score for an unhealthy lifestyle	−0.08*	−0.09*	− 0.0001	−0.04	− 0.04	−0.07	
8	Number of comorbidities	−0.10*	0.01	−0.01	−0.05	− 0.01	0.16*	− 0.03

Spearman’s Rho is presented

*e-MMM* estimated masseter muscle mass, *SMM* skeletal muscle mass

*p < 0.05

In both sexes, the SMM was significantly positively correlated with the e-MMM, maximum occlusal force, and gum score. The e-MMM was significantly positively correlated with the maximum occlusal force and gum score. The maximum occlusal force and gum score were significantly positively correlated. Age was significantly negatively correlated with the SMM, e-MMM, maximum occlusal force, and gum score and was significantly positively correlated with the number of comorbidities. On the other hand, the number of functional teeth was significantly positively correlated with the maximum occlusal force and gum score among males but correlated with only the gum score among females. The combined score for an unhealthy lifestyle was significantly negatively correlated with the e-MMM, gum score, and age in men and with the SMM and e-MMM in women.

### SEM

**Fig. 2:**
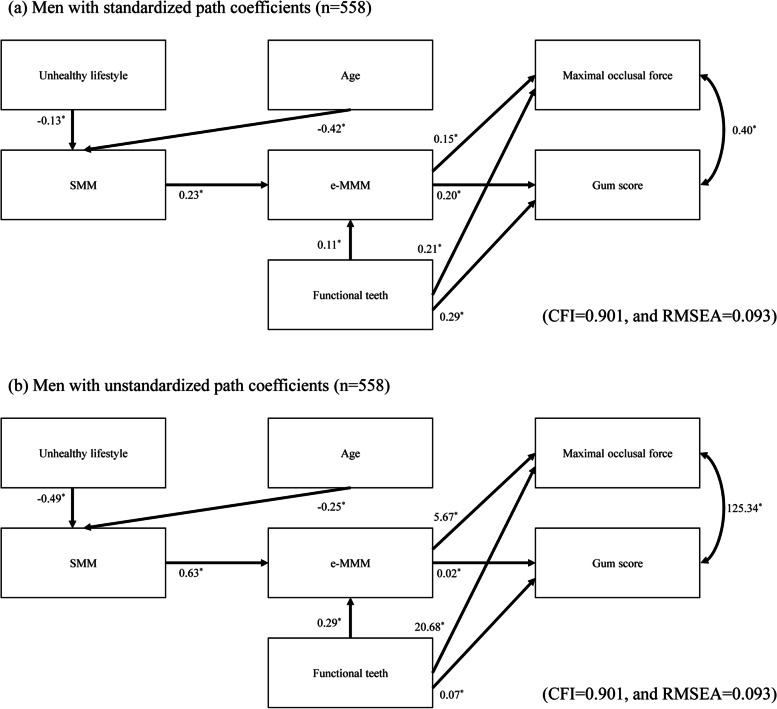
SEM of the relationships among SMM, e-MMM, and masticatory variables in men. **a** Model for men with standardized path coefficients (*n* = 558). **b** Model for men with unstandardized path coefficients (*n* = 558). ^*^p < 0.05. CFI=comparative fit index, RMSEA=root mean square error of approximation

**Fig. 3:**
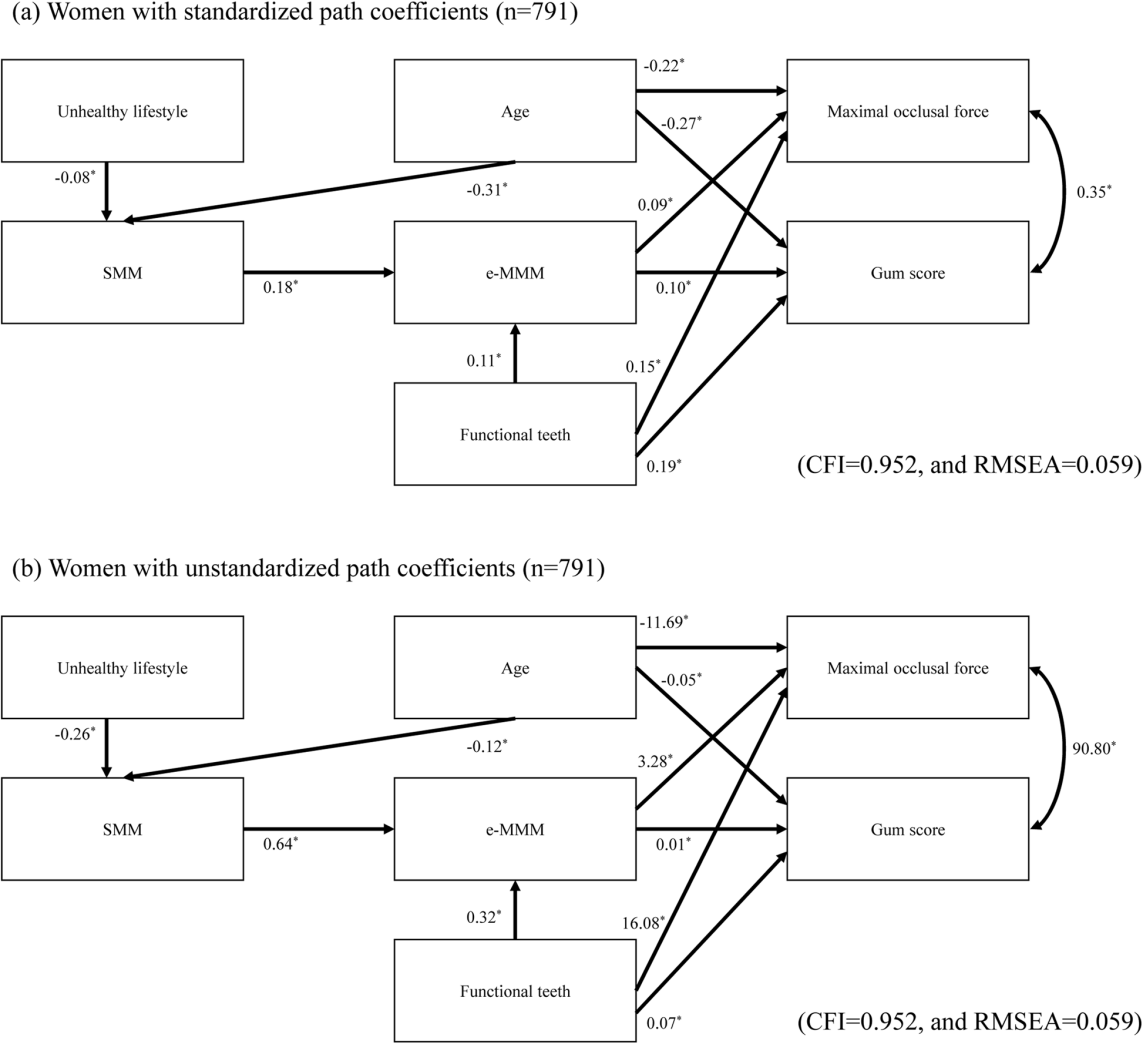
SEM of the relationships among SMM, e-MMM, and masticatory variables in women. **a** Model for women with standardized path coefficients (*n* = 791). **b** Model for women with unstandardized path coefficients (*n* = 791). ^*^p < 0.05. CFI = comparative fit index, RMSEA = root mean square error of approximation

The SEM was assessed separately for men (Fig. 2; CFI = 0.901, and RMSEA = 0.093) and women (Fig. 3; CFI = 0.952, and RMSEA = 0.059). In the figures, single-headed arrows with standardized (Figs. 2a and 3a) and unstandardized (Figs. 2b and 3b) path coefficients indicate the effects of the observed variables on the respective dependent variables. The double-headed arrows indicate correlations between measurement errors. In men and women, the direct path from the SMM to the e-MMM was significant, as was the direct path from the e-MMM to the maximum occlusal force and the gum score. We additionally confirmed that the indirect effect of the SMM on the maximum occlusal force and the gum score mediated by the e-MMM was significant (men; standardized coefficient [95% CI] = 3.64 [1.31 to 5.96] for the maximum occlusal force and 0.01 [0.01 to 0.02] for the gum score, women; 2.01 [0.38 to 3.81] for the maximum occlusal force and 0.01 [0.002 to 0.01] for the gum score, respectively).

The standardized coefficients were directly comparable. We found that the effect of the e-MMM on the gum score (standardized coefficient = 0.10) was comparable to that on the maximal occlusal force (standardized coefficient = 0.09) in women. This indicates that a reduction in masseter muscle mass could affect masseter muscle performance and strength to equal extents. On the other hand, the clinical meaning of a one-unit increase alone is presented with unstandardized coefficients. According to the unstandardized path coefficients presented in Figs. 2b and 3b, the maximal occlusal force is expected to increase by 5.7 N among men and by 3.3 N among women in response to a 1-cm^3^ increase in the e-MMM.

The number of functional teeth had a direct effect on the e-MMM, occlusal force, and gum score. The maximum occlusal force and the gum score were correlated with each other. Age had a significant direct effect on the SMM, maximum occlusal force, and gum score in women but had a significant direct effect only on the SMM in men.

Significant negative effects of an unhealthy lifestyle on the SMM were observed in men and women. The inclusion of comorbidities did not lead to a better-fitting model for men or women.

**Fig. 4:**
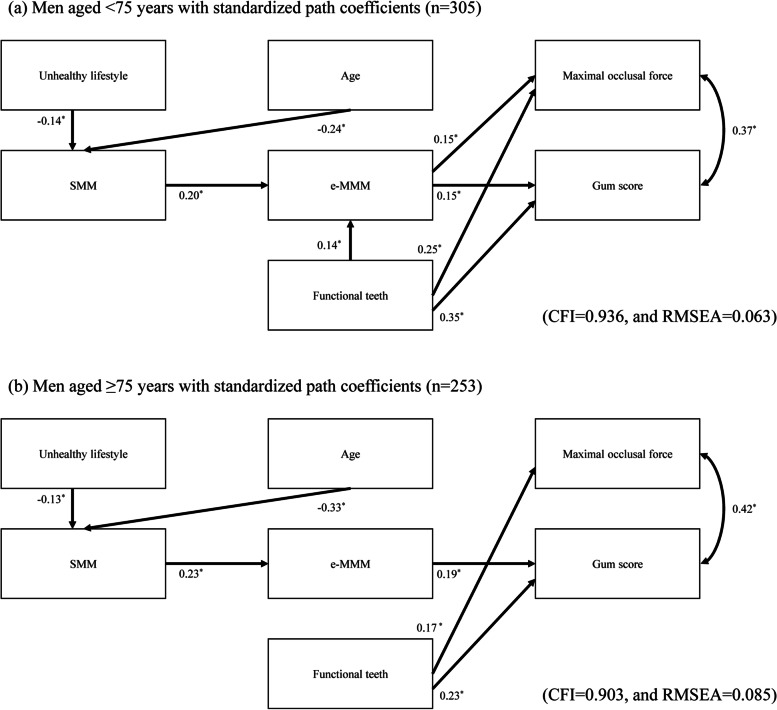
Age-stratified SEM of the relationships among SMM, e-MMM, and masticatory variables in men. **a** Model for men aged < 75 years with standardized path coefficients (*n* = 305). **b** Model for men aged ≥75 years with standardized path coefficients (*n* = 253). ^*^p < 0.05. CFI = comparative fit index, RMSEA = root mean square error of approximation

**Fig. 5:**
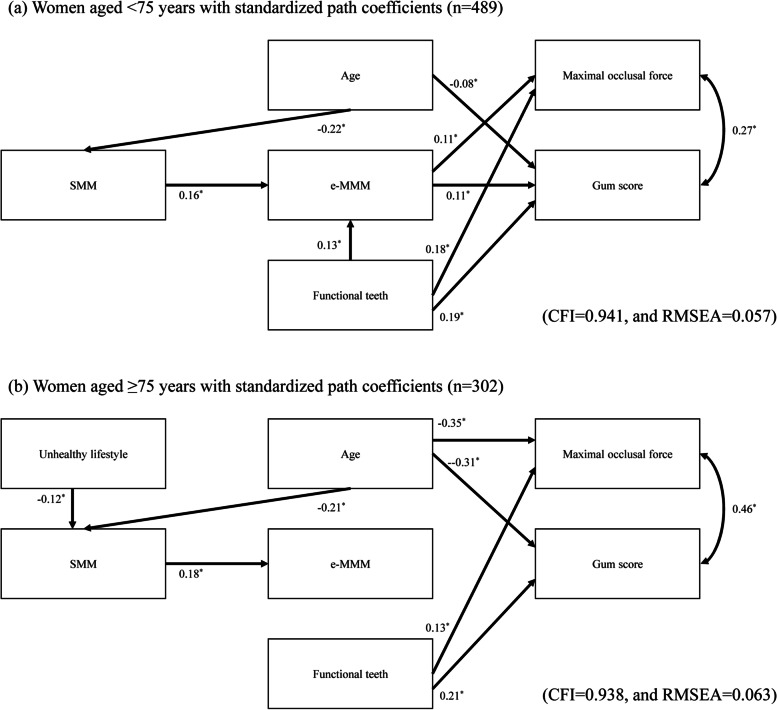
Age-stratified SEM of the relationships among SMM, e-MMM, and masticatory variables in women. **a** Model for women aged < 75 years with standardized path coefficients (*n* = 489). **b** Model for women aged ≥75 years with standardized path coefficients (*n* = 302). ^*^p < 0.05. CFI = comparative fit index, RMSEA = root mean square error of approximation

The results of the age-stratified analyses are presented in Figs. 4 and 5. The direct path from SMM to e-MMM remained significant regardless of age category or sex. The direct path from the number of functional teeth to oral function also retained significance regardless of age category or sex. In contrast, the effect of e-MMM on oral function became weak in both sexes of the group of participants ≥75 years old.

## Discussion

We assessed the e-MMM in community-dwelling older adults and investigated the associations of the e-MMM with the SMM, masseter muscle function assessed by the gum score, masseter muscle strength assessed by the maximal occlusal force, and the dentition status. Masseter muscle mass was significantly associated with masseter muscle function and strength. In addition, we observed that the SMM was significantly indirectly associated with the gum score and the maximal occlusal force and that the e-MMM mediated this association. The dentition status independently affected the e-MMM, the gum score, and the maximal occlusal force.

To our knowledge, this is the first study to simultaneously examine the associations among whole-body SMM; masseter muscle mass, function and strength; and dentition status in community-dwelling older adults and to elucidate the indirect effect of SMM on oral function through masseter muscle mass.

The present study has the following strengths. First, our study had a large sample size and included a considerable amount of contextual data, which enabled us to construct an SEM with sufficient information. Second, we first obtained the e-MMM from a large cohort of older adults. To date, masseter muscle mass assessment has not often been conducted in population-based surveillance. Magnetic resonance imaging (MRI) has been used to measure masseter muscle mass [28]. Although MRI-based masseter muscle mass (MRI-MMM) measurements are reliable and valid, this method requires many resources, including relevant equipment, trained examiners (manpower), and time. MRI scans are contraindicated for individuals with implanted sternal devices and other metal implants. These limitations may interfere with the common use of MRI-MMM measurements. Recently, we developed a more readily applied method of masseter muscle mass estimation based on muscle morphology and ultrasound-measured muscle thickness [8]. The masseter muscle mass estimated by this method was validated against the MRI-MMM, the gold standard, and found to be appropriate for masseter muscle mass measurement in large cohorts. We demonstrated that a low e-MMM was significantly associated with poor oral function (i.e., low masseter muscle performance and strength).

Etiological factors for the generalized loss of SMM include an age-related decline in hormone levels, increases in inflammation and oxidative stress, and lifestyle behaviors, including smoking and physical inactivity [29]. We observed a significant direct path from an unhealthy lifestyle to the generalized loss of SMM, which is consistent with previous findings [29]. The oral cavity is an organ of one body system. Etiological factors for the generalized loss of SMM may also affect masseter muscle mass. Previous studies have demonstrated that the loss of muscle mass is associated with the loss of strength [30] and functional impairment [14] in older adults. Another study demonstrated that masseter muscle thickness and occlusal force can simultaneously improve with chewing exercise [31]. Considering the above findings, it is biologically plausible that SMM has direct effects on e-MMM and that e-MMM has direct effects on masseter muscle strength and function, as shown in our study. The relationships among SMM and masseter muscle mass, strength, and function observed in this study are consistent with the findings of a previous study demonstrating significant individual associations among these health determinants, that is, associations between SMM and masseter muscle thickness [9, 10], between masseter muscle thickness and occlusal force [17, 32, 33], between masseter muscle tension and masticatory ability [34], between SMM and masticatory function [35, 36], and between SMM and occlusal force [35]. Our study provides one potential explanation for the observation in previous studies that older adults with sarcopenia have poor oral function [36, 37]. Furthermore, a hypothesized path in which a generalized loss of muscle mass and function is associated with a loss of swallowing muscle mass has been constructed based on previous epidemiological studies [11–13]. Our hypothesized model in which the whole-body SMM affects orofacial muscles is agreement with this speculation.

The direct path from the number of functional teeth to e-MMM was significant. Older adults with a low number of functional teeth tend to avoid foods that are hard to chew [38]. Hard-to-chew food avoidance can lead to low muscular activity during mastication. Low masseter muscle activity can cause muscle atrophy [9, 17], which manifested as low masseter muscle mass in this study. Direct paths from the number of functional teeth to e-MMM, occlusal force, and gum score were observed in our SEM, which have interesting clinical implications. The number of functional teeth describes the dentition status and includes both natural and prosthetic teeth. Therefore, both 1) preserving the natural teeth through adequate maintenance programs and regular professional hygiene care and 2) prosthodontic treatment to replace missing teeth have the potential to maintain or improve masseter muscle mass and function in older people regardless of their whole-body SMM. These possibilities should be investigated in future studies.

We did not observe a direct effect of age on the e-MMM in this study. The effect of aging may become significant in other parts of the body prior to affecting the masseter muscle. A previous study did not detect a significant association between masseter muscle thickness and age [9]. Our findings are in agreement with those from a previous study.

Although a hypothetical path was shown by our SEM, this study had a cross-sectional design, which prevented us from assessing the temporality of the association. Evaluating prospective associations among SMM, e-MMM, and masticatory function is an important next step. It should also be noted that some of the associations in our SEM might have had the opposite effect from that hypothesized. Oral health and functions play a significant role in nutrition. Low e-MMM can have a significant impact on SMM due to poor oral function and malnutrition. We did not obtain quantitative data on nutrition or diet and were therefore unable to test this hypothesis.

We detected a path from e-MMM to masseter muscle strength and function in our SEM, which indicated that the loss of masseter muscle mass occurs prior to the loss of masticatory function. Because impaired masticatory function is associated with malnutrition [39], measurement of masseter muscle mass can be an effective screening tool for persons who are at risk of developing malnutrition before their masticatory function declines to a clinically significant level. However, there is another interpretation of the association between masseter muscle mass and masticatory function. It is possible that muscle weakness and decreased function lead to diminished mastication activity, consequently leading to secondary muscular disuse atrophy. Therefore, low masseter muscle mass might be both the cause and result of the age-related loss of strength and function of the masseter muscle. From the age-stratification analyses, we observed that the effect of e-MMM on oral function was weak in older age group (≥ 75 years old). In older individuals, dentition status might be more important for oral function than masseter muscle mass. Additional longitudinal studies are needed to elucidate the interrelationships among masseter muscle mass, strength, and function with regard to aging.

The direct path from age to oral function (i.e., the gum score and the maximal occlusal force) was significant only in women. As presented in Table 1, we observed sex differences in the gum score and maximal occlusal force. Men had higher level of oral function than women. Researchers found a sex difference in physical performance among older community-dwelling Japanese individuals [40]. Similar to the case for oral function, men had a higher level of physical performance than women. The researchers also found age-related declines in all physical performance parameters that they studied. Furthermore, most physical performance parameters declined with age at a faster pace in women than in men. Considering our SEM results, the effect of aging on oral function could be greater in women than in men. The exact reason for this sex difference is unclear. Future studies to obtain further data such as muscle quality, maxillofacial morphology, and hormone levels are necessary to investigate the sex differences in age-related declines in oral function.

The present study has some limitations. First, our analyses did not distinguish the statuses of different types of prosthetic teeth, such as denture teeth, pontics on bridges, or implants. Based on our available dataset, it is not possible to count the number of artificial teeth separately for dentures, pontics, and implants. In addition, the model fit statistics of the SEM that included the numbers of both natural teeth and replaced teeth (sum of the numbers of artificial teeth in dentures, pontics, and implants) failed to reach the a priori-defined threshold. Although we obtained novel findings regarding the relationships among whole-body SMM, masseter muscle mass, and oral function, the effect of dentition status should be studied in more detail in the future. In addition, the length of time a tooth was missing and tooth mobility were not considered during dental examination. Therefore, we could not assess their effects on oral function. Second, the current study was designed to evaluate the masseter muscle quantitatively rather than qualitatively. A previous study showed that the loss of muscle mass only partly accounted for the loss of strength [30]. Muscle quality could be another important determinant of the loss of strength. Unfortunately, the effect of muscle quality on oral function could not be evaluated based on the available data. Third, the individuals included had voluntarily participated in the survey. Additionally, the present study population included only older Japanese adults, so the results may not be generalizable to other groups. Fourth, we did not examine masticatory muscles other than the masseter muscle. The masseter muscle plays a significant role in mastication [7, 8], and the Manual for Improvement of Oral Function issued by the Ministry of Health, Labor, and Welfare in Japan recommends that the masseter muscle is palpated in the evaluation of oral function [41]. However, the masseter muscle works in combination with other skeletal muscles in the head and neck region, including the temporal muscle and medial and lateral pterygoid muscles. Interrelationships among these muscles with regard to SMM and masticatory function should be studied in future work. Finally, we did not determine whether other potential confounders, such as neurological function, dietary intake, polypharmacy status, and physical function influenced the observed associations.

## Conclusions

Low SMM was significantly associated with poor oral function in older Japanese adults. Masseter muscle mass mediated the association between SMM and oral function. The dentition status was independently associated with oral function. Future studies evaluating the potential effect of interventions for SMM improvement and prosthodontic treatment on masticatory function in older adults with a low SMM may be of great interest.

## Data Availability

The data presented in this study are available upon request from the corresponding author. The data are not publicly available due to ethicolegal restrictions imposed by the Ethics Committee of the Tokyo Metropolitan Institute of Gerontology.

## Abbreviations

BIC: Bayesian information criterion
BMI: Body mass index
CFI: Comparative fit index
e-MMM: Estimated masseter muscle mass
MRI: Magnetic resonance imaging
MRI-MMM: MRI-based masseter muscle mass
RMSEA: root mean square error of approximation
SEM: Structural equation modeling
SMM: Skeletal muscle mass
